# Comparative Population Genetics in the Human Gut Microbiome

**DOI:** 10.1093/gbe/evab116

**Published:** 2021-05-24

**Authors:** William R Shoemaker, Daisy Chen, Nandita R Garud

**Affiliations:** 1 Department of Ecology and Evolutionary Biology, University of California, Los Angeles, California, USA; 2 Department of Human Genetics, University of California, Los Angeles, California, USA

**Keywords:** microbiome, population genetics, comparative population genetics, microbial evolution

## Abstract

Genetic variation in the human gut microbiome is responsible for conferring a number of crucial phenotypes like the ability to digest food and metabolize drugs. Yet, our understanding of how this variation arises and is maintained remains relatively poor. Thus, the microbiome remains a largely untapped resource, as the large number of coexisting species in the microbiome presents a unique opportunity to compare and contrast evolutionary processes across species to identify universal trends and deviations. Here we outline features of the human gut microbiome that, while not unique in isolation, as an assemblage make it a system with unparalleled potential for comparative population genomics studies. We consciously take a broad view of comparative population genetics, emphasizing how sampling a large number of species allows researchers to identify universal evolutionary dynamics in addition to new genes, which can then be leveraged to identify exceptional species that deviate from general patterns. To highlight the potential power of comparative population genetics in the microbiome, we reanalyze patterns of purifying selection across ∼40 prevalent species in the human gut microbiome to identify intriguing trends which highlight functional categories in the microbiome that may be under more or less constraint.

Significance

The human gut microbiome has unmatched potential to inform the field of comparative population genetics. However, the microbiome as a resource has yet to be fully tapped. In this review, we investigate that potential, highlighting features of the microbiome that make it unique venue for the study of comparative population genomics. We then build on these points by discussing how the seemingly complex nature of the microbiome can be viewed as an opportunity rather than a hinderance, a point that we convey by reexamining patterns of purifying selection in the human gut microbiome.

## Introduction

The human microbiome is a complex ecosystem composed of hundreds of interacting species. Although the diversity of the microbiome has been extensively studied at a species level, each species harbors genetic diversity that is quite varied across hosts as well as within a host over time (Zhu et al. 2010, 2019; Faith et al. 2013; Schloissnig et al. 2013). This genetic diversity can confer a number of crucial traits to microbes as well as their hosts, such as the ability to digest food, metabolize drugs, and evade antibiotics. However, our understanding of how these genetic variants arise and segregate via population genetic forces—for example, random genetic drift, mutation, recombination, selection, and migration—across the hundreds of species that call our guts home, is relatively nascent (Garud and Pollard 2020).

Our knowledge of how evolution proceeds in a community context is similarly underdeveloped. Much of our intuition about the evolution of microorganisms come from studying individual species in isolation of one other (Herron and Doebeli 2013; Lieberman et al. 2014; Tenaillon et al. 2016; Good et al. 2017; Xue et al. 2017; Bruger and Marx 2018). By contrast, the microbiome is composed of hundreds of interacting species and strains in which both ecological and evolutionary forces simultaneously act (Good and Hallatschek 2018; Garud and Pollard 2020). Specifically, changes in the genetic composition of resident lineages via the evolutionary processes of random genetic drift, mutation, selection, and recombination can occur on the same timescale as changes in strain frequencies via ecological processes. These simultaneous ecological and evolutionary processes within the human gut microbiome affords a unique opportunity: rather than studying one species at a time, we can study the population genetics and ecology of many (≳40) species almost simultaneously. Thus, the human gut microbiome is a model system for studying comparative population genetics across coexisting species in a natural environment.

Comparative population genetics is a rich area of study that has yielded insights into novel functional elements and population genetic processes in macroscopic organisms ranging from mammals (Davydov et al. 2010; Pollard et al. 2010; Lindblad-Toh et al. 2011; Romiguier et al. 2014) to *Drosophila melanogaster* (Clark et al. 2007; Lawrie and Petrov 2014). Now with the availability of hundreds of thousands of new genomes from the deep sequencing of commensal microbes (Almeida et al. 2019; Nayfach et al. 2019; Pasolli et al. 2019), similar comparative analyses can be made across microbial species and populations. However, unlike typical macroorganisms, the microbiome provides a rich opportunity to understand how ecological interactions between species modulate evolutionary dynamics within individual species.

## A Model System for Comparative Population Genetics: The Human Gut Microbiome

The human gut microbiome is a compelling system for comparative population genetics for several reasons. First, the short time scales on which evolutionary dynamics occur in the microbiome makes it possible to witness evolution in action as well as how evolution interacts with ecology (Garud et al. 2019; Poyet et al. 2019; Zhao et al. 2019; Roodgar et al. 2020; Yaffe and Relman 2020). Second, the meta-population structure of human microbiomes lends itself to treating measurements in spatially distant hosts as biological replicates, from which we can identify general principles. Third, given the massive amounts of data now available from thousands of individuals from around the world (Almeida et al. 2019; Pasolli et al. 2019), the time is ripe for the study of evolution in the microbiome. Fourth, the ability to experimentally manipulate this system (Roodgar et al. 2020) as well as its members (Barroso-Batista et al. 2014; Zhao et al. 2019; Ramiro et al. 2020) enables validation of computational predictions and discovery of new principles. Finally, the strong relevance of the microbiome to our health makes it a medically important system (Ley et al. 2005; Jakobsson et al. 2010; Duvallet et al. 2017; Thomas et al. 2019). Thus, comparative population genetic studies in the microbiome will not only elucidate general principles about the microbiome, but also the broader field of population genetics. Here we elaborate on these fundamental facets of the microbiome that make it an ideal system to study comparative population genetics in a naturally complex ecosystem.

### Rapid Evolution on Short Timescales

With relatively short generation times of ∼1–10 days in the human gut (Korem et al. 2015), microbes have the potential to rapidly evolve. This enables temporally resolved analyses that are almost impossible to replicate in macroscopic organisms. A sense of the evolutionary timescale of the human microbiome is best illustrated through a back-of-the-envelope calculation: given the low end of the range of generation times for the gut microbiome of ∼1 day (Korem et al. 2015; Milo and Phillips 2016), by the time a host reaches reproductive age, the microbiome can evolve on a timescale similar to that of the entire human species, roughly ∼10,000 generations (Moorjani et al. 2016; Scerri et al. 2018).

The ability to sample populations over a large number of generations can alter how evolutionary biologists approach their questions. Instead of relying predominantly on phylogenetic reconstruction from static data, researchers can effectively observe evolution in real time. Over the course of just a few months, new genotypes can emerge and recombine (Lin and Kussell 2017; Garud et al. 2019; Poyet et al. 2019; Zhao et al. 2019; Yaffe and Relman 2020; Zheng et al. 2020), and over extended time scales ultimately become lost or fixed. However, the necessary temporal resolution of sampling remains subject to the researcher’s question, as short-term adaptation in response to a temporary environmental perturbation (e.g., a host consuming antibiotics over a few weeks) may require denser sampling than in a constant environment.

### Replication across Hosts

That the human gut microbiome can be viewed a quasi-closed system holds implications for the investigation of evolutionary dynamics. Specifically, the physical separation of the guts of different hosts means that each host can be treated as a replicate evolution experiment, an observation that has been summarized by the captivating moniker “seven billion microcosms” (Lieberman 2018). This level of replication can be leveraged to identify targets of positive selection that recurrently accumulate fixation events in independent hosts (i.e., parallel evolution; Xue et al. 2017; Bertels et al. 2019; Lieberman et al. 2014; Zhao et al. 2019; Poyet et al. 2019). By combining large cohort sizes with temporally resolved sampling we can also examine how these signatures of parallelism change over time, allowing us to dissect the temporal dynamics of adaptation (Barroso-Batista et al. 2014). For example, targets of rapid adaptation typically harbor a disproportionate number of sites with strong beneficial fitness effects, leading to the fixation of multiple mutations within a short period of time (i.e., “coupon collecting”; Good et al. 2017). Alternatively, if the fitness effects of sites in a gene depend on whether prior mutations have fixed elsewhere in the genome, then the time between fixation events will be large (i.e., historical contingency; Gould 1990; Blount et al. 2008). The benefits of large cohorts are not limited to detecting adaptation. With large cohorts, we can also observe deleterious alleles segregating at extremely low frequencies that are likely subject to purifying selection (Lawrie and Petrov 2014). Combined with exciting recent theoretical developments (Neher and Shraiman 2012; Nicolaisen and Desai 2012; Cvijović et al. 2018; Good 2020), the replication across human gut microbiomes lends itself naturally to examining the evolutionary dynamics of purifying selection.

We note that whether replicate observations can, or need to, be treated as independent samples likely depends on the researcher’s question. If a researcher is examining evolutionary dynamics where identity-by-descent is a cofounding variable, they may wish to exclude species where the assumption of independence is unlikely to hold (e.g., extensive gene flow eroding spatial structure). For other analyses, high rates of gene flow among a set of species may serve as a benefit, providing replicate observations from which migration can be inferred.

### Ecology and Evolution Frequently Interact

It is becoming increasingly clear that mutations in many microbial populations do not fix or become extinct, and instead remain at intermediate frequencies for extended periods of time (Good et al. 2017; Good and Hallatschek 2018). This “strain” level structure constitutes a form of ecology that exists below the taxonomic level of species, and is commonly found within hosts for most gut microbiota (Truong et al. 2017; Garud et al. 2019). The sheer prevalence of strain structure in the human microbiome and the fact that they can differ on the order of a few nucleotides (Goyal et al. 2021) suggests that ecological and evolutionary dynamics occur on similar timescales in microbial systems, contrary to the historical belief that evolutionary timescales are longer than ecological timescales (Slobodkin 1980). For example, strain frequencies can fluctuate on the same time scale on which they acquire new genetic adaptations (Garud et al. 2019; Zhao et al. 2019). This observation, along with the relative ease with which a large number of species can be sampled across hosts, suggests that the human gut microbiome is a system with unmatched potential for the exploration of eco-evolutionary dynamics.

The presence of overlapping ecological and evolutionary timescales in the microbiome has spurred empirical and theoretical efforts to characterize eco-evolutionary interactions within the gut. A prime example is a recent mathematical model that describes how the frequency of a strain can change over time due to the acquisition of de novo mutations that affect the consumption of environmentally supplied resources and overall fitness (Good et al. 2018). Thus, evolution can affect ecological competition between strains with resource consumption as a mediating factor, which can ultimately alter community composition and structure. However, it is unlikely that eco-evolutionary interactions within the gut can be sufficiently captured by accounting for environmentally supplied resources alone. Rather, microorganisms often secrete secondary metabolic compounds, supplying additional resources that can be consumed by other species (i.e., cross-feeding; Fritts et al. 2021; Carlson et al. 2018). This metabolic dependency promotes species coexistence and becomes increasingly likely in communities with many species, an ecological bedrock that supports subsequent coevolution (D’Souza et al. 2018; Lilja and Johnson 2019). The widespread nature of this phenomenon may explain empirical patterns where it appears as though the arrow of causation between evolution and ecology is reversed, a prominent example being that the evolutionary diversification rate of a species is correlated with the number of species in its community (Madi et al. 2020).

### Experimental Manipulation

Although a natural system enables researchers to study complex phenomena that cannot be recapitulated exactly in the laboratory, some degree of experimental manipulation is necessary to validate predictions and generate new insights. Over the last few years, substantial progress has been made toward characterizing the evolutionary dynamics of adaptation in laboratory microbial populations. For example, lineage tracking via mass barcoding has allowed the distribution of fitness effects of individual de novo mutations to be inferred in certain species (Levy et al. 2015). As for the long-term dynamics of adaptation, theoretical progress has primarily been driven by contributions from statistical physics (Tsimring et al. 1996; Rouzine et al. 2003; Desai and Fisher 2007; Fisher 2013), where adaptation can be modeled as a distribution of fitness values that increase over time as existing variants fix and de novo mutations arise (i.e., the staircase model; Good and Hallatschek 2018). This theoretical progress was leveraged alongside lineage tracking to demonstrate that the traveling wave is an appropriate model of microbial adaptation over an extended timescale (Nguyen Ba et al. 2019), a conclusion that may be pertinent for understanding the adaptive dynamics of strains within the human gut microbiome.

The utility of these experimental tools is not restricted to the ecology-free single-species cases. For example, gene deletion via transposon mutagenesis libraries have been particularly effective for identifying loci that confer a growth advantage in environments with different resources (Cain et al. 2020) and different sets of cooccurring species (Thibault et al. 2019). These experiments have also been useful for species isolated from the gut (Ruiz et al. 2013) and in the gut microbiome of model organisms (Powell et al. 2016; Zimmermann et al. 2019; Barreto et al. 2020; Barroso-Batista et al. 2020; Ludington and Ja 2020). Thus, gene deletion experiments can serve as a compliment to traditional comparative population genetic analyses, allowing us to test hypotheses formed from metagenomic observational studies. Although all these studies fall short of true in vivo manipulation of human guts, they are useful approximations that allow for high-throughput manipulations to be performed and evolutionary and ecological hypotheses to be tested.

### Relevance to Health

The human microbiome is tightly associated with human health. The species composition of the gut microbiome is known to be essential for proper immunological (Belkaid and Hand 2014), neurological (Yano et al. 2015), and metabolic development (Rowland et al. 2018), and is associated with a number of human diseases including colorectal cancer, diabetes (Vallianou et al. 2018), and obesity (Ley et al. 2005, 2006). Although the connection of the human microbiome to host health has been primarily studied at the species level, genetic variants in the microbiome play a crucial role for health as well. Specifically, microbiome genetic variants can confer a number of critical traits to human hosts, including the digestion of new foods (Hehemann et al. 2010; Kenny et al. 2020), antibiotic resistance (Gautam et al. 2018), and the metabolization of drugs (Spanogiannopoulos et al. 2016). A comparative genomics approach will enable the discovery of new microbiome genetic variants (Sberro et al. 2019), which may ultimately be useful for the future development of effective microbiome therapies.

## Lessons from Comparative Population Genetics in the Microbiome

Although our understanding of the microbiome and the discipline of comparative population genetics have rapidly expanded since the emergence of next-generation sequencing almost two decades ago, their intersection is relatively recent. Therefore, the potential of comparative population genetics in the microbiome is still being realized. Here we present three goals for the future of comparative population genetics: the need to identify 1) previously unknown functional elements of microbial genomes, 2) evolutionary dynamics common to all species in the gut microbiome, and 3) individual species and genomic features that deviate from general patterns.

### Inference of Functionality

Currently, the annotation of genes in the microbiome and our understanding of their functionality is severely lacking, with an estimated 40% being “hypothetical” (Almeida et al. 2019). Comparative population genetics has the potential to shed light on the functions of existing hypothetical genes and assist with the identification of new ones. Much of the utility of comparative population genetics derives from the neutral theory of molecular evolution, which predicts that if mutations in functional regions of genomes tend to be deleterious, those regions will evolve at a slower rate than effectively neutral nonfunctional regions (Kimura 1983). This constraint allows for conserved elements of the genome to be identified between highly diverged species; a “phylogenetic footprint” (Lawrie and Petrov 2014). Using this basic assumption, comparative genomic analyses across groups of macroorganisms as diverse as *Drosophila* and mammals have yielded insight into novel proteins (Clark et al. 2007; Lawrie and Petrov 2014). Now, recent efforts have been made to apply this approach to the microbiome (Fremin and Bhatt 2020). Specifically, Sberro et al. (2019) recently performed a comparative analysis on shotgun microbiome metagenomic data and discovered thousands of novel small genes. Among their discoveries was a novel small ribosome-associated protein that seems to be transcribed and translated at high levels. Despite the fundamental functional significance of this protein, it may have been missed due to the historical focus on model organisms such as *E. coli* and common pathogens.

However, there is additional justification to claim that microorganisms harbor a substantial number of unannotated functional elements. Population genetic theory coupled with cellular energetics predicts that the vast majority of unannotated genes within the gut microbiome likely play some functional role (Lynch and Marinov 2015; Martinez-Gutierrez and Aylward 2019). For example, a single nonfunctional nucleotide within a microbial genome is visible to purifying selection (Lynch and Marinov 2015), a stark contrast to macroorganisms where junk DNA is highly prevalent (Lynch 2007). Coupled with the higher gene density in microorganisms due to overlapping open reading frames (Johnson and Chisholm 2004), this prediction suggests that the gut microbiome is a particularly apt system for researchers who wish to leverage statistical evidence provided by comparative population genomics to confirm the purported functionality of a given gene. Indeed, researchers are likely already acting on this prediction, as recent efforts combined comparative genomics with RNA-seq to identify ∼2,000 novel structural RNAs in the microbiome (Fremin and Bhatt 2020). With hundreds of species harboring genomes with high gene density across billions of hosts, the gut microbiome is still very much a proverbial “gold rush” for the discovery and characterization of novel proteins and RNAs.

### Robust Evolutionary Patterns

Arguably, it is necessary to gain some degree of knowledge regarding the typical evolutionary dynamics of a species in a given system before comparisons between species can be performed. At first glance, it would appear as if the goal of identifying general evolutionary patterns in the human gut microbiome is hopeless. There are few cases where population geneticists would say that we have sufficient knowledge of the evolutionary dynamics of one species, much less hundreds or thousands of species that interact in the same environment. Operating under this assumption, we would conclude that the complexity of the microbiome is irreducible. This is not entirely an unjustified claim, since if one is interested in the evolutionary dynamics of an individual species, how can those dynamics be sufficiently characterized if you cannot examine an isolated species in vivo?

The fault here is the idea that we need to understand the dynamics of individual species to understand the general dynamics of the system. Instead, progress can be made by temporarily abandoning the Cartesian framework that is ubiquitous in traditional biology (Levins and Lewontin 1987) and embracing an alternative approach, where we exchange determinism for a statistical property, the average over an ensemble of species. This rationale is essentially what physicists realized in the 19th century (Pathria and Beale 2011) and has been applied in recent years to examine the ecological dynamics of microorganisms, through the development of mathematical models (Barbier and Arnoldi 2017; Advani et al. 2018) as well as the investigation of empirical data (Descheemaeker and de Buyl 2020; Grilli 2020; Ji et al. 2020). It stands to reason that comparative population genetics could learn from such an approach. Although our argument here is primarily statistical, a spiritually similar argument has been made regarding the use of effective models that coarse-grain over taxonomic details to identify quantitative patterns in microbial ecology and evolution (Good and Hallatschek 2018).

Ultimately it is necessary to take stock of the set of patterns that remain robust across phylogenetically diverged species within the human gut, allowing us to identify the evolutionary dynamics that universally occur. Here, we will briefly examine a few notable evolutionary patterns that have been observed across species.

#### Population Structure

The genetic composition of commensal bacteria varies considerably from host to host (Schloissnig et al. 2013; Costea et al. 2017; Truong et al. 2017), suggesting that bacteria do not rampantly migrate between hosts. Instead, for each species, hosts are typically colonized by a handful of strains that seem to be unique to each host (Schloissnig et al. 2013; Garud et al. 2019). The typical number of strains within a species is variable, likely reflecting the degree that strains can diverge and evolve sufficient ecological differences necessary to coexist (Good et al. 2018). Though this within-host population structure does not seem to necessarily have bearing on across-host population structure, as the global biogeography of genetic variants can vary considerably across species. For example, the genetic diversity of *Eubacterium rectale* mirrors the genetic diversity of hosts (Nayfach et al. 2016; Costea et al. 2017; Truong et al. 2017; Tett et al. 2019; Karcher et al. 2020), whereas species such as *B. vulgatus* seem to show little or no geographic structure. The mechanisms responsible for variation in the global biogeography of species remains unclear. Vertical transmission from parents to infants may contribute, as strains from certain species are more likely to colonize and persist in infant guts, the genera *Bacteroides* and *Bifidobacterium* being noted examples (Lou et al. 2021). Though the benefit of being the first to colonize a host is likely temporary, as the majority of strains are replaced over several decades (Garud et al. 2019). Alternative mechanisms for varied levels of biogeography include traits that promote airborne transmission being restricted to certain lineages (Brown 2000) which may explain the variation in transmission rates among species (Brito et al. 2019), the interaction of the microbiome with the genetics of its host (Goodrich et al. 2016), and even the presence of spatial structure itself (Tropini et al. 2017), which may promote the preservation of genetic variation (Pearce and Fisher 2019), though these hypotheses remain to be fully tested.

#### Recombination

Although all bacteria reproduce clonally, the degree to which bacteria recombine varies widely (Smith et al. 1993; Vos and Didelot 2009). The recombination rate of a species determines whether populations evolve primarily via changes in genotype frequencies over time or as changes in the frequencies of individual alleles that are effectively independent (Neher and Shraiman 2011), which can have consequences for whether gene-specific versus genome-wide selective sweeps are more common (Shapiro et al. 2012; Bendall et al. 2016; Shapiro 2016;). To quantify recombination in bacteria, researchers have begun to characterize the statistical association of alleles at different loci (i.e., linkage disequilibria), where the degree of association can be viewed a function of the recombination rate. For several species found in the gut, as well as environmental samples and pathogens, linkage disequilibria tends to decay as the genetic distance between a pair of loci increases, which suggests that recombination may be common (Lin and Kussell 2017; Garud et al. 2019; Crits-Christoph et al. 2020; Sakoparnig et al. 2021). Such rampant recombination suggests that although microbes reproduce clonally, the label “asexual” is a misnomer. Instead, microorganisms are increasingly being deemed as “quasi-sexual,” where a large number of loci evolve independently instead of as genotypes despite the clonal nature through which they are reproduced (Smith et al. 1993; Rosen et al. 2015; Shapiro 2016). However, observed levels of linkage disequilibrium tend to be higher than what is expected under free recombination for many species (Garud et al. 2019). Some species may be truly clonal (Smith et al. 1993; Vos and Didelot 2009), whereas others are likely subject to additional evolutionary forces that can generate correlations between sites, such as demographic history and selection. Selection seems to play a particularly prominent role, where recent developments in population genetic theory provide the groundwork necessary for subsequent empirical investigation (Arnold et al. 2020; Good 2020). These forces will need to be disentangled to understand the full extent of recombination in the microbiome.

#### Short-Term Evolution within Hosts

Recently, it was found that evolutionary changes can occur in the human gut microbiome on short timescales of just a few months and even days (Ghalayini et al. 2018; Garud et al. 2019; Poyet et al. 2019; Zhao et al. 2019; Roodgar et al. 2020; Yaffe and Relman 2020), and that strain replacements are generally rare over that timescale. These evolutionary changes modify the haplotypes of existing lineages and seem to derive from a mixture of de novo mutations and horizontal gene transfer via recombination. The recombination-seeded events are a unique mode of adaptation that highlight how a complex community can maintain a reservoir of adaptive genetic material, which may be particularly useful in rapidly fluctuating environments where evolution via de novo mutations may take a long time. Thus, complex communities may be able to modulate the mode and tempo of evolution of focal species (Frazão et al. 2019; Madi et al. 2020). At longer time scales, these evolutionary changes tend to give way to ecology, as strain replacements become common. These seemingly contrasting dynamics will ultimately require researchers to intuit what evolutionary and ecological dynamics are relevant on a given timescale and, ultimately, construct models that bridge separate dynamics.

#### Purifying Selection

**Fig. 1.**
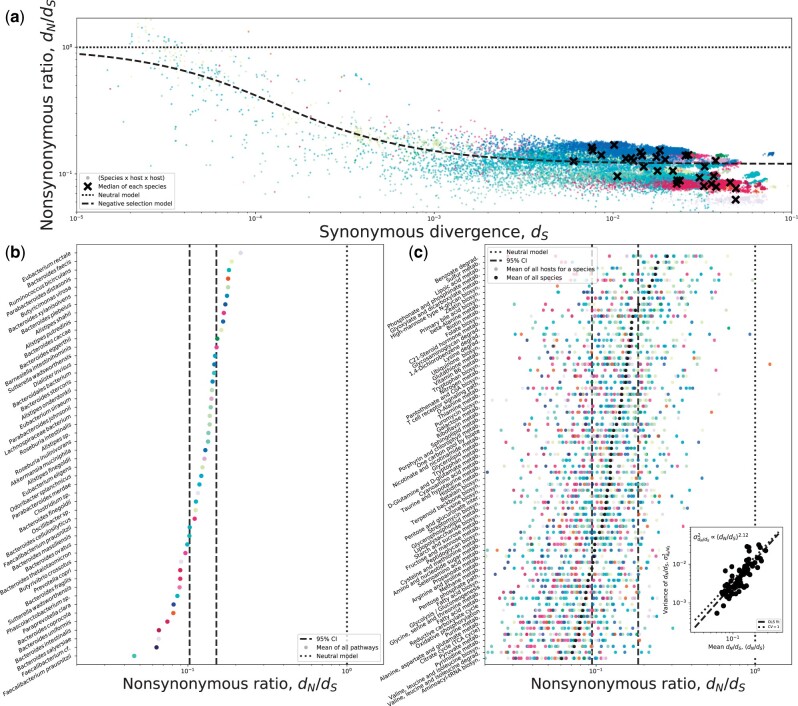
(*a*) The relationship between synonymous divergence on the *x* axis ($d_{S}$) and the ratio of nonsynonymous and synonymous divergences ($d_{N}/d_{S})$ on the *y* axis follows the form predicted by purifying selection across species (Eq. S8 from S1D in Garud et al. 2019). Though by color coding individual species, we see that data points tend to be grouped by species identity, where certain species fall above or below the prediction. (*b*) By grouping genes by their pathways and generating an appropriate null distribution via permutation, we can identify pathways that, under the assumptions of the model, are under stronger or weaker purifying selection than expected by chance. We can then examine how the mean $d_{N}/d_{S}$ ($〈d_{N}/d_{S}〉$) of a given pathway relates to its variance ($\sigma_{d_{N}/d_{S}}^{2}$), where the variance increases slightly faster than the square of $〈d_{N}/d_{S}〉$, suggesting that the coefficient of variation is greater than one (inset figure in *b*). (*c*) By inverting our permutation scheme, we can identify the set of species that are subject to stronger or weaker purifying selection than expected by chance.

By comparing haplotypes of a given species from different hosts, we can focus on patterns that are the outcome of evolutionary dynamics that have operated over an extended timescale. One such pattern is how the ratio of nonsynonymous to synonymous divergences ($d_{N}/d_{S}$) changes as synonymous divergence ($d_{S}$) increases, which would indicate in what direction selection tends to dominate over an extended timescale. Looking at empirical data from the microbiome, it is clear that $d_{N}/d_{S}$ tends to decrease with increasing $d_{S}$, suggesting that purifying selection tends to dominate as lineages diverge (fig. 1*a*; Garud et al. 2019). Surprisingly, the shape of the relationship can be captured by an effective model of selection composed of two parameters: a single selection coefficient and the fraction of sites subject to selection (Garud et al. 2019). Some species clearly have values of $d_{N}/d_{S}$ that are further from this prediction than others, an observation that we will return to below. But as a first approximation we can say that purifying selection explains genome-wide patterns of genetic divergence across species within the gut (fig. 1*a*).

### Identifying Deviations from General Trends

It may not be clear how a researcher can leverage universal evolutionary patterns to identify genes and species of interest. Indeed, interest in identifying exceptional species and targets of evolution is likely why many researchers compare species in the first place (Leffler et al. 2012; Lawrie and Petrov 2014; Huber et al. 2020). Here, we will re-examine the relationship between $d_{S}$ and $d_{N}/d_{S}$ in figure 1*a* to illustrate how starting with a strong universal pattern can provide a backdrop against which we identify deviations from the overall trend. We see that certain species tend to fall above or below the prediction of an effective model of purifying selection (fig. 1*a*). To identify species that are subject to stronger or weaker purifying selection than expected by chance, we first coarse-grain genes by their annotated metabolic pathways, providing a set of variables shared across species. We can then permute species-level observations within a given pathway and establish 95% confidence intervals, a nonparametric test that allows us to identify species with exceptional $d_{N}/d_{S}$ (fig. 1*b*).

Though we coarse-grained genes by necessity, it allowed us to perform additional analyses in which we leverage information across multiple species. First, we can see that certain pathways typically have lower $d_{N}/d_{S}$ than others, suggesting that they are subject to stronger purifying selection (fig. 1*c*). We can then identify pathways that are subject to stronger or weaker purifying selection than expected by chance by permuting values of $d_{N}/d_{S}$ across pathways within each species and establishing confidence intervals (see supplementary information, Supplementary Material online). The results of this test align with our biological intuition: essential pathways tend to be under stronger purifying selection (e.g., glycolysis, nucleotide biosynthesis, Krebs cycle, etc.), whereas pathways that rely on specific resources (e.g., sulfur metabolism) tend to be under relatively relaxed selection—an observation consistent with prior analyses using polymorphism data (Schloissnig et al. 2013).

Because we have observations from many species, we can continue our comparative population genetic analyses and examine the statistical properties of $d_{N}/d_{S}$ across pathways. First, we can determine whether the relative spread of $d_{N}/d_{S}$ remains similar across pathways by examining whether the ratio of the standard deviation to the mean (i.e., the coefficient of variation) remains constant. We find that this is the case, as the mean $d_{N}/d_{S}$ of a pathway ($〈d_{N}/d_{S}〉$) across species is linear with respect to its variance ($\sigma_{d_{N}/d_{S}}^{2}$; fig. 1*b*). This observation is reminiscent of Taylor’s Law (Taylor 1961), a pattern often found in ecological systems (Grilli 2020). Similar to Taylor’s Law, we find that the slope of this relationship is not significantly different from two (t-test=0.684; P=0.248), which we can interpret as the mean being equal to the standard deviation across pathways (coefficient of variation $=\sigma_{d_{N}/d_{S}}^{2}/{〈d_{N}/d_{S}〉}^{2}=1$). This observation suggests that the relative dispersion of $d_{N}/d_{S}$ remains constant as the overall level of constraint within a pathway is relaxed. Though values of $d_{N}/d_{S}$ across pathways are not independent, as the correlation in $d_{N}/d_{S}$ across pathways for a given pair of species tends to decay with phylogenetic distance (*β* = −0.104, $P<10^{-6}$), suggesting that the strength of purifying selection within a given essential pathway is moderately conserved through evolutionary time. However, this does not provide an explanation of why certain pathways are subject to stronger purifying selection than others, or why the strength of selection varies across lineages, a question that can likely only be answered by incorporating additional biological details about the pathways and species themselves (Bielawski and Yang 2004; Aguileta et al. 2009). Rather, it illustrates how investigating deviations from an empirical pattern can lead to novel findings.

## Future Directions for Comparative Population Genetics in the Microbiome

The study of comparative population genetics in the human microbiome is nascent and full of potential. There are multiple avenues of progress in the microbiome field that will benefit comparative population genetics as a discipline.

First, advances in sequencing technology will allow us to refine our estimates of important quantities. For example, long-read technologies, such as nanopore metagenomic sequencing, will allow researchers to quantify linkage between physically distant sites (Yaffe and Relman 2020; Zlitni et al. 2020; Bharti and Grimm 2021; Karst et al. 2021), providing higher resolution to uncover fundamental evolutionary processes of recombination, mutation, and adaptation within and across species. These advances, coupled with decreasing costs of library preparation (Baym et al. 2015), will allow researchers to sample large cohorts over time and observe how genotypes dissipate into alleles and reemerge via recombination over their sojourn times, enabling us to build more detailed evolutionary models.

Second, the physical environment of the human gut creates unique evolutionary pressures that are interesting avenues of study in their own right. Temperature differences between the inside of the gut and the outside environment (Groussin and Gouy 2011) as well as spatial structure (Tropini et al. 2017; Bradburd and Ralph 2019) can affect the evolutionary dynamics of microbial species. Even deceptively simple features of the gut such as its physical resemblance to a chemostat (Locey and Lennon 2019) or the peristaltic mixing that arises due to digestion can produce complex ecological and evolutionary dynamics (Cremer et al. 2016).

Finally, there is arguably as much a need to examine the phenotypic effects of molecular evolution as there is to characterize molecular evolutionary dynamics. The genes that contribute to adaptation in the microbiome ultimately encode physical aspects of microbes that may impact human phenotypes. This means that subsequent experiments will be necessary to gain a more concrete understanding of the dynamics of adaptation in the gut and their phenotypic relevance (Lynch et al. 2014; Lynch and Trickovic 2020).

Future computational, statistical, theoretical, and experimental advances in the already exciting field of comparative population genetics in the human microbiome will generate insights that span multiple disciplines, generate fundamental theory that may be relevant to comparative genomics in other systems, and may even ultimately have the potential to inform human health.

## Supplementary Material

Supplementary data are available at *Genome Biology and Evolution* online.

## Supplementary Material

evab116_Supplementary_Data
